# Emergence of criticality in models of real neurons

**Published:** 2026-09-08

**Authors:** David P. Carcamo, Christopher W. Lynn

**Affiliations:** 1 Department of Physics, Yale University, New Haven, CT 06520, USA; 2 Quantitative Biology Institute, Yale University, New Haven, CT 06520, USA; 3 Wu Tsai Institute, Yale University, New Haven, CT 06520, USA

## Abstract

Critical systems sit near boundaries between qualitatively distinct behaviors. When inferring models of neural activity, this proximity to criticality is thought to require the precise tuning of parameters. Here, we show that as the number of neurons increases, criticality can emerge naturally without fine-tuning. When computing observable statistics from parameters (the forward problem), some small regions in parameter space map to large regions in statistics space. These special parameters are precisely those near criticality. Thus, when inferring parameters from experimental measurements (the inverse problem), models concentrate near critical points, and this concentration becomes stronger as the system grows. We illustrate this flow toward criticality across many large-scale recordings in the mouse brain. In the Curie-Weiss model of Ising spins, we find that all of the recordings collapse to a first-order phase transition, despite substantial differences in the underlying systems. Together, these results suggest a resolution to the tension between criticality and fine-tuning in models of neural activity.

Biological networks—from flocks of birds and schools of fish to clusters of bacteria and genetic interactions—often exhibit signatures of criticality.^1–5^ In the brain, populations of neurons spanning different animals and experimental settings produce avalanches of cascading activity.^6–9^ The correlations between neurons extend over spatiotemporal scales,^10–13^ follow heavy-tailed distributions,^14–17^ and display scale-free structure.^18^ Models inferred from neural data consistently lie near phase transitions, where small changes in model parameters produce large changes in collective activity.^17, 19–23^ Moreover, these models appear to move closer to criticality as the number of neurons increases.^17, 19–21^ In simulations, this proximity to criticality provides functional advantages, from improved sensitivity to optimized information processing.^24–27^

However, in the space of possible models, critical points occupy a vanishingly small volume.^1, 20, 28^ This suggests that criticality requires the precise calibration of parameters, forcing the system to delicately balance between distinct regimes.^29^ This fine-tuning stands in stark contrast to the incredible diversity of neural systems across species, regions, and cell types. Significant efforts to address this tension have produced mechanisms for self-organization as well as alternative explanations for signatures of criticality.^16, 29–37^ Yet the question remains: In models of real neurons, does criticality require fine-tuning?

To answer this question, we examine the geometry of the mapping between experimental measurements and models of neural activity.^38, 39^ Although critical models occupy small regions in the space of parameters, as the number of neurons grows, these models map to large regions in the space of observable statistics. Thus, as experiments record from larger populations, many different measurements from a range of systems collapse to similar models near criticality.

## Minimal mapping from experiments to models

To investigate this collapse in large populations of neurons, we focus on the simplest experimental measurements: the average activity and the strength of correlations. For a system of N neurons (i=1,…,N), the state of the population at a given instant is defined by a binary vector $x=\left\{x_{i}\right\}$, where each neuron is either active ($x_{i}=1$) or silent ($x_{i}=-1$). The instantaneous activity of the population is $\mu =\frac{1}{N}\sum_{i}x_{i}$, and experiments give us access to the mean activity $m=\frac{1}{N}\sum_{i}\left\langle x_{i}\right\rangle$, where ⟨⋅⟩ denotes an experimental average. Similarly, the total strength of correlations is given by $\chi =\frac{1}{N}\sum_{i,j}\left\langle x_{i}x_{j}\right\rangle -\left\langle x_{i}\right\rangle \left\langle x_{j}\right\rangle =N\left(\left\langle \mu^{2}\right\rangle -m^{2}\right)$. The different combinations (m,χ) define our space of statistics, in which each point represents the possible outcome of an experiment. In Fig. 1a, we illustrate these statistics for 146 separate recordings from $N\approx 10^{3}$ to $10^{4}$ neurons in mice: 100 electrophysiological recordings from thalamus and visual cortex, ^40,41^ 45 calcium-imaging recordings from visual cortex, ^42^ and one calcium-imaging recording from hippocampus. ^43,44^ These experiments span a large region in the space of statistics, with different activities *m* and nearly two orders of magnitude in correlations χ.

Every model of activity defines a distribution over collective states *P*(***x***), but there are infinitely many models consistent with a given set of statistics (*m, χ*). To break this degeneracy, the only distribution justified by the measurements alone, without introducing any additional structure, is the one with maximum entropy,

(1) $$P(x)=\frac{1}{Z}\exp\left[h\sum_{i}x_{i}+\frac{\lambda }{2N}{\left(\sum_{i}x_{i}\right)}^{2}\right],$$

where *Z* is the normalizing partition function (Methods).^45, 46^ The parameters *h* and *λ* are Lagrange multipliers that must be computed so the model matches the observed statistics *m* and *χ*, respectively. This inference procedure defines a unique mapping from experimental measurements (*m, χ*) to model parameters (*h, λ*).

Maximum entropy models such as Eq. (1) have been applied across a wide range of neural systems to study the minimal consequences of experimental measurements.^17, 19–22, 47^ In the context of recurrent neural networks, *P*(*x*) is the steady-state distribution for a system of logistic neurons with biases determined by *h* and all-to-all interactions with strength determined by *λ*.^48, 49^ In statistical physics, it is the Curie-Weiss model, which is a special case of the Ising model of interacting magnetic spins.^21, 28, 50^ Importantly, the critical behavior of the Curie-Weiss model is known exactly. In the thermodynamic limit *N* → ∞, the system undergoes a second-order phase transition at the point (*h, λ*) = (0, 1). This is the classic Ising transition from disorder (*λ* < 1) to order (*λ* > 1) via spontaneous symmetry breaking.^28, 51^ Additionally, the system exhibits a line of critical points at *h* = 0 and *λ* > 1 that define a first-order phase transition.^21^ Crossing this line leads to a discontinuous jump from negative activity *m* (for *h* < 0) to positive activity (for *h* > 0). Together, the second-order point and the first-order line represent the only critical models within the entire two-dimensional space of parameters.

Although we have defined a unique mapping between statistics (*m, χ*) and parameters (*h, λ*), evaluating this map is computationally challenging. In maximum entropy models, calculations require averaging over all 2^N^ states, which typically restricts applications to populations of *N* ~ 100 neurons using Monte Carlo simulations.^19^ Here, given the simplicity of the Curie-Weiss model, we only need to sum over the *N* + 1 discrete values of the population activity *μ*, weighted by their multiplicities (Methods). This allows us to calculate exact statistics (*m, χ*) and numerically invert the model to infer exact parameters (*h, λ*) for systems of up to *N* ~ 10^4^ neurons.^21^

Applying this method to each of the recordings, we arrive at 146 separate models (Fig. 1b). In each of these models, we find that *h* < 0, such that silence is favored over activity (*m* < 0), and *λ* > 0, such that neurons are positively correlated. Despite substantial differences in the measured statistics, brain regions, population sizes, experimental setups, and recording modalities, the inferred models collapse onto a narrow region in the space of parameters (Fig. 1b, *inset*). Specifically, the different recordings are all described by similar models near criticality, with *h* ≈ 0 and *λ* ≥ 1. This collapse reinforces signatures of criticality that have been discovered in many distinct experiments and models of neural activity.^6–23^ By contrast, if we shuffle the activity of each neuron to destroy correlations, then the statistics become similar while the models, which are no longer critical, remain distinct (Supplementary Information).

## Geometry of the forward map

To understand how disparate systems collapse to criticality, we must first understand the geometry of the forward map from model parameters to observable statistics. For a given number of neurons *N*, each set of parameters (*h, λ*) defines a specific model [Eq. (1)], from which we can compute statistics (*m, χ*) (Methods). Consider the region of parameters outlined in Fig. 2a, which defines a manifold of models with different statistics. This mapping from parameters to statistics is highly nonlinear, with parameters near the critical line expanding to large volumes in the space of statistics and parameters far from criticality contracting to small volumes (Fig. 2b). This nonlinearity becomes even more dramatic as the number of neurons increases, with a large number of possible statistics captured only by a small number of critical models (Fig. 2b, *right*).

To quantify the strength of this expansion and contraction, we evaluate the shift in statistics induced by small changes in parameters. For example, the fluctuation-dissipation relation reveals that the response in activity *m* from a perturbation in *h* is dictated by the strength of correlations, such that $\frac{\partial m}{\partial h}=\chi$. At the critical point (*h, λ*) = (0, 1), the correlation *χ* diverges as the number of neurons *N* increases. Thus, for models near criticality, small changes in *h* sweep over many different activities *m*, particularly as the population grows.

More generally, as parameters map to statistics, the scaling of volume is defined by the determinant of the Jacobian,

(2) $$|J|=\left|\begin{array}{ll} \frac{\partial m}{\partial h} & \frac{\partial m}{\partial \lambda }\\ \frac{\partial \chi }{\partial h} & \frac{\partial \chi }{\partial \lambda } \end{array}\right|,$$

which, for simplicity, we refer to as the Jacobian.^38, 39^ In the Curie-Weiss model, we can calculate |*J*| exactly, even for large *N* (Methods). As the external field h increases, tending toward the critical line, the Jacobian grows monotonically (Fig. 2c). Far from criticality, volume contracts (|*J*| < 1), while close to criticality, volume expands (|*J*| > 1). Moreover, even if we hold parameters fixed near the critical line, as the number of neurons increases, the Jacobian grows by orders of magnitude (Fig. 2d). Thus, when mapping from parameters to statistics, not all models are created equal; a handful expand to cover many possible observations, and these are precisely the models near criticality.

## Growth induces flow toward criticality

When we say that criticality requires fine-tuning, we are implicitly placing a prior on the space of parameters. If this prior is uniform, then finding a model near criticality is surprising. However, if we define models based on their statistics, then this prior becomes highly non-uniform, as demonstrated in Fig. 2b. Instead, one should select a prior that is invariant under reparameterization. This is Jeffreys prior, which, for maximum entropy models, is proportional to the square root of the Jacobian |*J*| (Methods).^39, 52^ Even for small systems, the Jacobian is largest near the critical line, and this maximum sharpens as the number of neurons increases (Fig. 3a). From this perspective, models near criticality should become orders of magnitude more likely as populations grow.^38^

We arrive at the same conclusion by examining the geometry of the inverse map from statistics to parameters. As *N* increases, we have seen that parameters near criticality expand to cover many statistics (Fig. 2b). Conversely, as experiments grow, we should expect many distinct measurements to contract toward critical models, a phenomenon previously observed in financial data.^38^ We can investigate the contraction quantitatively by treating the number of neurons *N* as a free parameter in the model [Eq. (1)]. Holding the statistics (*m, χ*) fixed, increasing *N* induces a flow in the parameters (*h, λ*), which we compute using the Hubbard-Stratonovich transformation (Methods). For interactions below the critical strength (*λ* < 1), we find that increasing *N* leads to a decrease in *λ*, pushing models toward independence (Fig. 3b). By contrast, for strongly interacting systems (*λ* ≥ 1), the external field *h* increases as the population grows, pushing models toward the critical line. This flow to criticality can be studied using thermodynamic quantities such as the susceptibility and specific heat (Supplementary Information).^20^ Here, we are able to investigate the flow directly by defining a grid of statistics (*m, χ*), which represents a collection of possible experiments (Fig. 3c, *inset*). For small *N*, the inferred models span a relatively large region of parameters (Fig. 3c, *left*). Yet as the number of neurons increases, even with the statistics held fixed, many of the inferred models become indistinguishable as they contract to criticality (Fig. 3c, *right*).

Ultimately, we seek to understand whether the same collapse occurs in neural activity. For each of the recordings in Fig. 1, we subsample the population at increasing sizes *N*. For each of these subpopulations, we measure the activity *m* and correlation *χ*, and then infer the corresponding parameters (*h, λ*). For small *N*, correlations are weak, and the activity is nearly independent (Fig. 4a, *left*). As the populations grow, correlations become stronger, and the recordings occupy an expanding volume of different statistics (Fig. 4a). In stark contrast, the corresponding models contract into a shrinking volume of similar parameters (Fig. 4b). Across all populations, the interactions rise above the critical strength *λ* ≥ 1, and the external fields approach *h* = 0. Thus, despite expanding in the space of statistics, all of the recordings contract toward criticality in the space of models.

## Thermodynamic collapse

As the number of neurons increases, each individual interaction in the model becomes weaker, replaced by the average influence of a growing population. Thus, as experiments grow, one might expect a reduced mean-field theory to emerge.^21^ Here, we show that, while the simplest version of this idea fails, an improved theory captures the mapping between measurements and models. In doing so, we find that an entire two-dimensional region of statistics collapses to the one-dimensional critical line in the thermodynamic limit *N* → ∞.

Since the probability of each collective state *x* depends only on the population activity *μ*, the model can be written P(x)∝exp⁡[-Nf(μ)], where *f*(*μ*) is the free energy per neuron (Methods). In the limit *N* → ∞, this free energy has a unique minimum for any parameters (*h, λ*) not on the critical line. In turn, this minimum dominates any average over *P*(*x*), leading to the traditional mean-field theory,^53^ with the average activity defined self-consistently by m=tanh⁡(λm+h) and correlations by $\chi =\frac{1-m^{2}}{1-\lambda \left(1-m^{2}\right)}$. As a direct consequence, for any activity *m*, mean-field theory sets a hard upper bound on the strength of correlations (Methods).^21^ However, when examining the neural recordings, we see that all of the measured correlations break this bound, some by multiple orders of magnitude (Fig. 5a). So where has the map between models and experiments broken down?

We have failed by focusing on models that are not critical. Consider the hippocampal recording.^43, 44^ For small subpopulations, the free energy maintains one global and one local minimum, but as *N* increases, these minima become degenerate (Fig. 5b). This is the defining feature of a first-order phase transition. In order to map between parameters and statistics, we must therefore include both minima in any average over *P*(*x*). The resulting double-well approximation predicts that, for measured statistics (*m, χ*), the corresponding parameters are given by

(3) $$h=\frac{1}{Nm_{0}}\tanh^{-1}\left(\frac{m}{m_{0}}\right)\;\text{and}\;\lambda =\frac{1}{m_{0}}\tanh^{-1}\left(m_{0}\right),$$

where $m_{0}=\sqrt{m^{2}+\chi /N}$ (Methods). We compare this prediction with the exact parameters inferred from different recordings (Fig. 5c,d). As populations grow, the double-well approximation becomes more accurate, eventually converging to the true parameters for *N* ≳ 100 neurons.

Upon inspection, the double-well approximation reveals something striking. For a given set of statistics (*m, χ*), the inferred interactions exceed the critical strength (*λ* ≥ 0). Then, as the number of neurons increases, the external field vanishes (*h* → 0), pushing models toward the critical line (Fig. 5e). This is precisely the flow that we observe in the neural recordings (Fig. 4b). Thus, in the thermodynamic limit *N* → ∞, we find that a large region of possible statistics—which includes many different measurements of neural activity—collapses directly to the line of criticality.

## Discussion

The brain exhibits several signatures associated with models poised near criticality.^6–23^ How can such a diverse array of behaviors—across species, systems, and scales—correspond to such a narrow range of models? To answer this question, we study on the geometry of the map between experimental measurements and models of neural activity. Although critical models occupy a vanishingly small volume in the space of parameters, as the number of neurons grows, these models expand to cover a large volume of observable statistics.^38^ Thus, rather than requiring fine-tuning, criticality can emerge naturally in models of large-scale activity.

Focusing on the average activity *m* and correlation strength *χ*, the Curie-Weiss model defines the minimal mapping from experiments to models. Due to this simplicity, the critical behavior of the model is defined unambiguously, and we can exactly compute the mapping between statistics and parameters in very large populations (Methods). Although the Curie-Weiss model fails to capture many features of neural activity,^21^ we expect our central findings to hold much more broadly. For any statistics measured in experiments, the maximum entropy principle defines the most unbiased mapping to models.^46, 47^ In maximum entropy models, as the number of neurons increases, first-order phase transitions are defined by discontinuities in the statistics, and second-order phase transitions are defined by divergences in the Jacobian.^19, 28, 38^ If we expect parameters to flow toward models where the Jacobian is large, then, quite generally, we should expect experimental measurements to contract toward criticality. Even in more complex descriptions of neural dynamics,^23, 29, 54^ where criticality may not be defined, inferred models should concentrate where small changes in parameters produce large changes in behaviors. Investigating this contraction in different models and biological networks is an exciting direction.

Finally, we note that the brain can exhibit signatures of criticality without itself being critical. Indeed, some of these signatures can arise through correlated responses to latent variables or timevarying inputs.^34–37^ Even defining criticality for a system as complex as the brain remains an open challenge.^8^ However, when we fit models to neural data, our results suggest that criticality may not require fine-tuning. Instead, as experiments advance to record larger populations, many distinct measurements may collapse to similar models, thus providing simplified descriptions of neural systems.

## Methods

### Neural data.

We analyze neural activity from 146 electrophysiological and calcium-imaging recordings in previous experiments. The recordings were selected to be as large as possible, with no other selection criteria. The thalamus and visual cortex data were recorded electrophysiologically during a visual change-detection task as part of the Allen Institute Visual Behavior Neuropixels dataset.^40, 41^ We analyze 100 recordings, each containing *N* = 1, 705 ± 355 (mean ± SD) neurons from different brain regions. For each recording, spikes were sorted into 10 ms time bins, which then defines the binary activity of each neuron. The visual cortex data were recorded using two-photon calcium imaging by Stringer *et al*.^42^ The data consist of 45 recordings from seven awake mice, with *N* = 10, 506 ± 1, 737 neurons per recording (mean ± SD). Neural activity was measured at a sampling rate of approximately 1.5 Hz while mice ran on an air-floating ball. Visual stimuli included natural images, distorted natural images, drifting gratings, and gray screens used to measure spontaneous activity. The hippocampal data were recorded using two-photon calcium imaging by Gauthier and Tank.^44^. The population consists of 1,485 neurons recorded at 30 Hz from the CA1 region while the head-fixed mouse ran along a virtual track. In the calcium imaging data, fluorescence traces for each neuron were binarized into active or inactive states.

### Maximum entropy model.

Across all datasets, we represent the activity of neuron i at time t by a binary variable $x_{i}(t)\in \{-1,1\}$, where $x_{i}(t)=1$ corresponds to activity and $x_{i}(t)=-1$ to silence. The collective state of the population is then defined by a binary vector $x=\left\{x_{i}\right\}$. For a recording of length T, we measure the average activity of each neuron $\left\langle x_{i}\right\rangle =\frac{1}{T}\sum_{t}x_{i}(t)$ and the correlation between each pair of neurons $\left\langle x_{i}x_{j}\right\rangle =\frac{1}{T}\sum_{t}x_{i}(t)x_{j}(t)$. From these measurements, we then compute the population-level average $m=\frac{1}{N}\sum_{i}\left\langle x_{i}\right\rangle$ and correlation = $\frac{1}{N}\sum_{ij}\left\langle x_{i}x_{j}\right\rangle -\left\langle x_{i}\right\rangle \left\langle x_{j}\right\rangle$.

The only model *P*(*x*) that is justified by these measurements, with no other sources of structure, is the one with maximum entropy.^45–47^ This distribution takes the form of the Curie-Weiss model in Eq. (1), where

(4) $$Z=\sum_{x}\exp\left[h\sum_{i}x_{i}+\frac{\lambda }{2N}{\left(\sum_{i}x_{i}\right)}^{2}\right]$$

is the normalizing partition function. The parameters *h* and λ must be inferred so that the model matches the experimental activity *m* and correlation *χ*, respectively.

### Exact statistics and inference.

The probability of each state only depends on the instantaneous population activity $\mu =\frac{1}{N}\sum_{i}x_{i}$. When taking model averages, this allows us to replace the intractable sum over states *x* with a tractable sum over the *N* + 1 distinct values of *μ*, weighted by the number of states for each *μ*. The partition function is given by

(5) $$Z=\sum_{\mu }C\left(N,\frac{1+\mu }{2}N\right)\exp\left(Nh\mu +N\frac{\lambda }{2}\mu^{2}\right),$$

where *C*(·, ·) is the binomial coefficient. Similarly, the average activity and correlation strength can be efficiently computed using

(6) $$m=\frac{1}{Z}\underset{\mu }{\sum }C\left(N,\frac{1+\mu }{2}N\right)\mu \;\text{exp}\left(Nh\mu +N\frac{\lambda }{2}\mu^{2}\right),$$

(7) $$\chi =\frac{N}{Z}\underset{\mu }{\sum }C\left(N,\frac{1+\mu }{2}N\right)\mu^{2}\;\text{exp}\left(Nh\mu +N\frac{\lambda }{2}\mu^{2}\right)-Nm^{2}.$$

Eqs. (5)–(7) provide an exact solution for the forward map from parameters to statistics in very large populations.

To solve the inverse map from statistics to parameters, we note that at fixed *m*, there is a one-dimensional manifold of models along which *χ* varies, with parameters *h*(λ) and λ.^21^ Parameter changes along this manifold satisfy

(8) $$dm=\frac{\partial m}{\partial \lambda }d\lambda +\frac{\partial m}{\partial h}dh=0,$$

so that

(9) $$\frac{dh(\lambda )}{d\lambda }=-{\left(\frac{\partial m}{\partial h}\right)}^{-1}\frac{\partial m}{\partial \lambda }=-\frac{1}{\chi }\frac{\partial m}{\partial \lambda }.$$

We numerically integrate this equation starting from the independent model *h*(0) = tanh^−1^ (*m*) until the model reaches the target value of *χ*. In combination with Eqs. (5)–(7), this provides an exact solution to the inverse map.

### Jacobian, Jeffreys prior, and Fisher information.

The Jacobian in Eq. (2) is defined by the derivatives,

(10) $$\frac{\partial m}{\partial h}=N\left(\langle \mu^{2}\rangle -m^{2}\right)=\chi ,$$

(11) $$\frac{\partial m}{\partial \lambda }=\frac{N}{2}\left(\langle \mu^{3}\rangle -m\langle \mu^{2}\rangle \right),$$

(12) $$\frac{\partial \chi }{\partial h}=N^{2}\left(\langle \mu^{3}\rangle -3m\langle \mu^{2}\rangle +2m^{3}\right),$$

(13) $$\frac{\partial \chi }{\partial \lambda }=\frac{N^{2}}{2}\left(\langle \mu^{4}\rangle -{\langle \mu^{2}\rangle }^{2}-2m\langle \mu^{3}\rangle +2\langle \mu^{2}\rangle m^{2}\right).$$

Note that each of the above model averages can be computed efficiently using the method described previously. Jeffreys prior defines the distribution over models that is invariant under changes in parameters $p(h,\lambda )\propto \sqrt{\left|I(h,\lambda )\right|}$, where I(h,λ) is the Fisher information matrix.^39, 52^ For the Curie-Weiss model, the Fisher information is given by^38^

(14) $$I=N^{2}\left(\begin{array}{cc} \left\langle \mu^{2}\right\rangle -\langle \mu \rangle^{2} & \frac{1}{2}\left(\left\langle \mu^{3}\right\rangle -\left\langle \mu^{2}\right\rangle \langle \mu \rangle \right)\\ \frac{1}{2}\left(\left\langle \mu^{3}\right\rangle -\left\langle \mu^{2}\right\rangle \langle \mu \rangle \right) & \frac{1}{4}\left(\left\langle \mu^{4}\right\rangle -{\left\langle \mu^{2}\right\rangle }^{2}\right) \end{array}\right).$$

Note that the determinant of the Fisher information is proportional to that of the Jacobian, $\left|I|=\frac{N}{2}|J\right|$. Thus, Jeffreys prior is proportional to the square root of the Jacobian.

### Hubbard-Stratonovich transformation.

In order to treat the number of neurons *N* as a free parameter in the model, we use the Hubbard-Stratonovich transformation.^55^ Replacing interactions in the model with an integral over a fluctuating field, we can rewrite the partition function exactly as,

(15) $$Z=\sqrt{\frac{N}{2\pi \lambda }}2^{N}\int d\psi e^{-Nl\left(\psi \right),}$$

where

(16) $$l(\psi )=\frac{\psi^{2}}{2\lambda }-\mathrm{ln cosh}(h+\psi ).$$

For any choice of *h*, λ, and *N*, the statistics can be computed numerically by evaluating the integrals,

(17) $$m=\frac{1}{\int d\psi e^{-Nl(\psi )}}\int d\psi e^{-Nl(\psi )}\text{tanh}(h+\psi ),$$

(18) $$\begin{array}{c} \chi =1-Nm^{2}+(N-1)\frac{1}{\int d\psi e^{-Nl(\psi )}}\int \tanh^{2}(h+\psi )e^{-Nl(\psi )}. \end{array}$$

### Parameter flow with increasing system size.

The system size now appears as a continuous variable, allowing us to differentiate the statistics with respect to N. Letting $\langle \cdot \rangle_{\psi }$ denote an average over the fluctuating field $P(\psi )\propto e^{-Nl(\psi )}$, we have

(19) $$\frac{\partial m}{\partial N}=m\langle l(\psi )\rangle_{\psi }-\langle \tanh(h+\psi )l(\psi )\rangle_{\psi },$$

(20) $$\frac{\partial \chi }{\partial N}={\left\langle \tanh(h+\psi {)}^{2}\right\rangle }_{\psi }+(N-1)\left({\left\langle \tanh(h+\psi {)}^{2}\right\rangle }_{\psi }\langle l(\psi )\rangle_{\psi }-{\left\langle \tanh(h+\psi {)}^{2}l(\psi )\right\rangle }_{\psi }\right)-m^{2}-2Nm\frac{\partial m}{\partial N}.$$

To understand how models flow when the number of neurons increases, we hold *m* and *χ* fixed while allowing *h* and λ to vary with *N*. Taking the total derivative with respect to *N* gives

(21) 0=ddNmχ=JddNhλ+∂∂Nmχ.

Increasing the number of neurons therefore induces the following flow in the space of parameters,

(22) ddNhλ=-J-1∂∂Nmχ.

In Fig. 3b, we plot the flow in parameters with respect to fractional changes in system size,

(23) ddln⁡Nhλ=-NJ-1∂∂Nmχ.

### Mean-field approximation.

To understand the thermodynamic limit *N* → ∞, we write the model as a function of the population activity $P(x)\propto e^{-Nf(\mu )}$, where

(24) $$f(\mu )=-h\mu -\frac{\lambda }{2}\mu^{2}-\frac{1}{N}logC\left(N,\frac{1+\mu }{2}N\right)$$

is the free energy per neuron. As the system becomes large, the free energy approaches

(25) $$f(\mu )=-h\mu -\frac{\lambda }{2}\mu^{2}+\frac{1+\mu }{2}\log\left(\frac{1+\mu }{2}\right)+\frac{1-\mu }{2}\log\left(\frac{1-\mu }{2}\right).$$

For parameters (h,λ) not on the critical line, the free energy has a unique minimum. In the limit N→∞, this minimum dominates any average over P(x), yielding the mean-field theory with m=tanh⁡(λm+h) and $\chi =\frac{1-m^{2}}{1-\lambda \left(1-m^{2}\right)}$.^53^ This theory places an upper bound on the strength of correlations,^21^

(26) $$\chi_{\max}(m)=\frac{m\left(1-m^{2}\right)}{m-{tanh}^{-1}(m)\left(1-m^{2}\right)}.$$

All of the recordings break this bound, some with correlations orders of magnitude stronger than allowed under mean-field theory (Fig. 5a).

### Double-well approximation.

The mean-field approximation fails to capture real neural activity because it only applies to models for which the free energy has a unique minimum; that is, non-critical models. When inferring models from neural recordings, the free energy develops two degenerate minima (Fig. 5b), the defining feature of a first-order phase transition.^21^ For large *N*, when taking averages over *P* (***x***), we must include both minima.

For λ>1 and h≈0, the free energy has two minima $\mu_{\pm }\approx \pm m_{0}$, where $m_{0}$ is the positive solution to the mean-field equation with no external field, $m_{0}=tanh\left(\lambda m_{0}\right)$. Only including these minima, the average activity is given by

(27) $$m=\frac{\mu_{+}e^{-Nf\left(\mu_{+}\right)}+\mu_{-}e^{-Nf\left(\mu_{-}\right)}}{e^{-Nf\left(\mu_{+}\right)}+e^{-Nf\left(\mu_{-}\right)}}=\frac{m_{0}e^{Nhm_{0}}-m_{0}e^{-Nhm_{0}}}{e^{Nhm_{0}}+e^{-Nhm_{0}}}=m_{0}\tanh\left(Nhm_{0}\right).$$

Differentiating with respect to h yields the correlation $\chi =N\left(m_{0}^{2}-m^{2}\right)$. Finally, inverting these equations provides the solution to the inverse map from statistics to parameters in Eq. (3).

## Supplementary Material

1

Supplementary text and figures accompany this paper.

## Figures and Tables

**Fig. 1 |:**
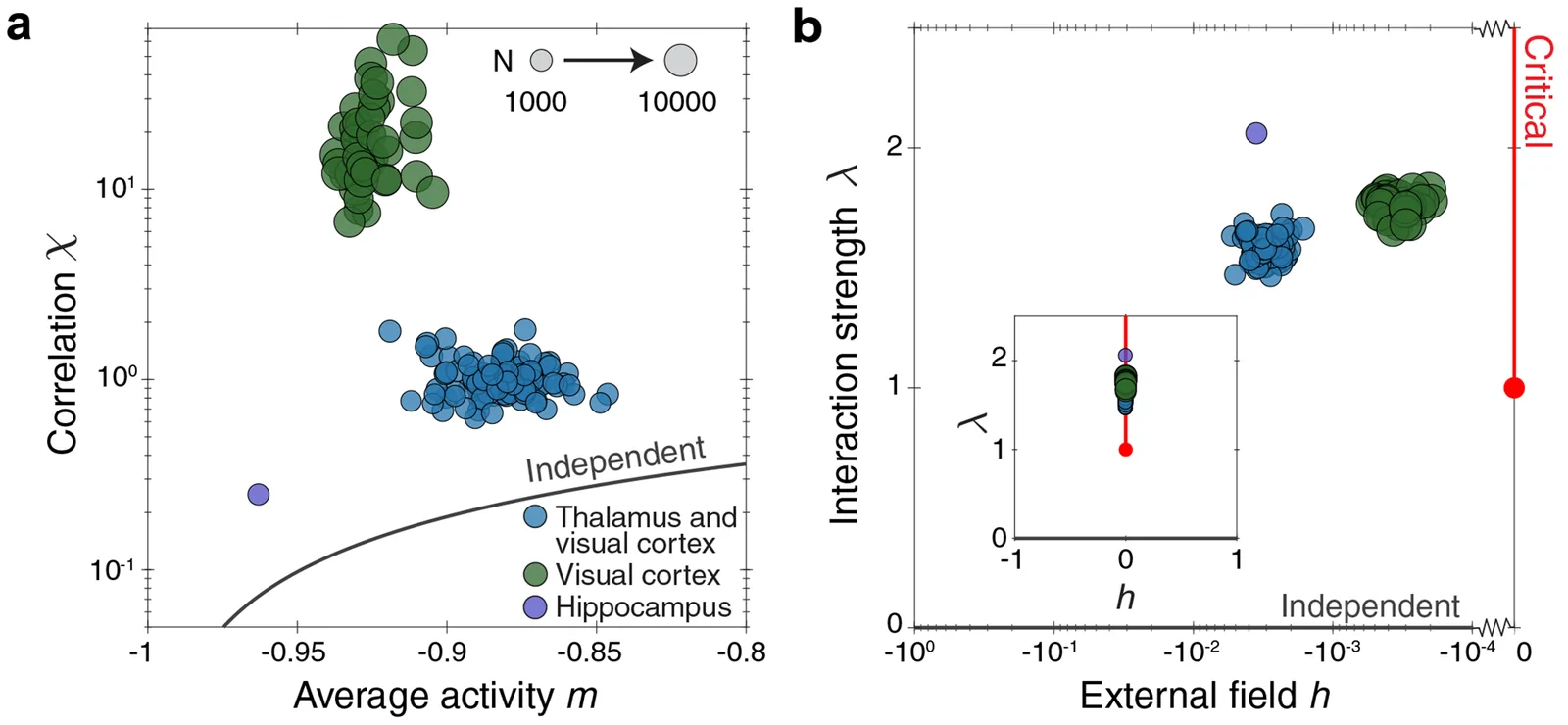
Neural activity maps to models near criticality. **a**, Average activity *m* and correlation strength *χ* for 146 recordings of neuronal activity: 100 electrophysiological recordings from thalamus and visual cortex (blue),^40, 41^ 45 calcium-imaging recordings from visual cortex (green),^42^ and one calcium-imaging recording from hippocampus (purple).^43, 44^ Marker size indicates the number of neurons *N* in each population. Gray line represents statistics that map to independent models (with λ = 0), defined by *χ* = 1 – *m*^2^. **b**, Inferred Ising parameters *h* and λ for each of the recordings in **a**. Red line defines the set of critical models in the thermodynamic limit *N* → ∞, and gray line represents independent models (λ = 0). Inset displays the same parameters with a linear *h* axis, illustrating the collapse of the different recordings to critical models.

**Fig. 2 |:**
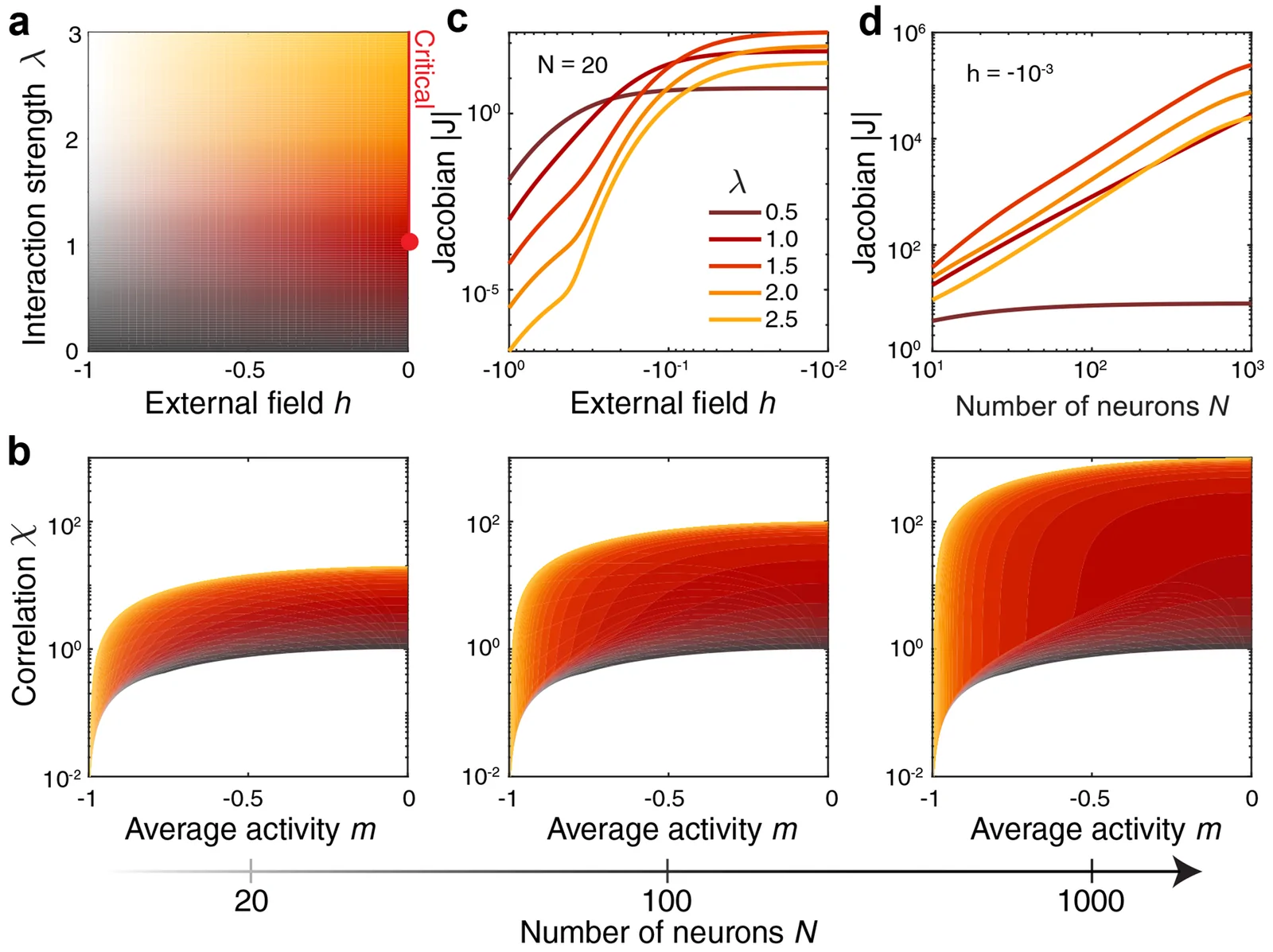
Expansion and contraction from parameters to statistics. **a**, Region in the space of parameters with color indicating the interaction strength λ and saturation indicating the external field *h*. Red line represents criticality in the thermodynamic limit. **b**, Regions in the space of statistics covered by the parameters in **a** for increasing numbers of neurons *N*. **c**, Scaling factor defined by the determinant of the Jacobian |*J*| increases monotonically as a function of the external field *h*. Each line represents *N* = 20 neurons interacting with a different strength λ. **d**, Jacobian |*J*| as a function of the system size *N* at fixed *h* = −10^−3^. For models near criticality (λ ≥ 1), the Jacobian grows dramatically with the number of neurons.

**Fig. 3 |:**
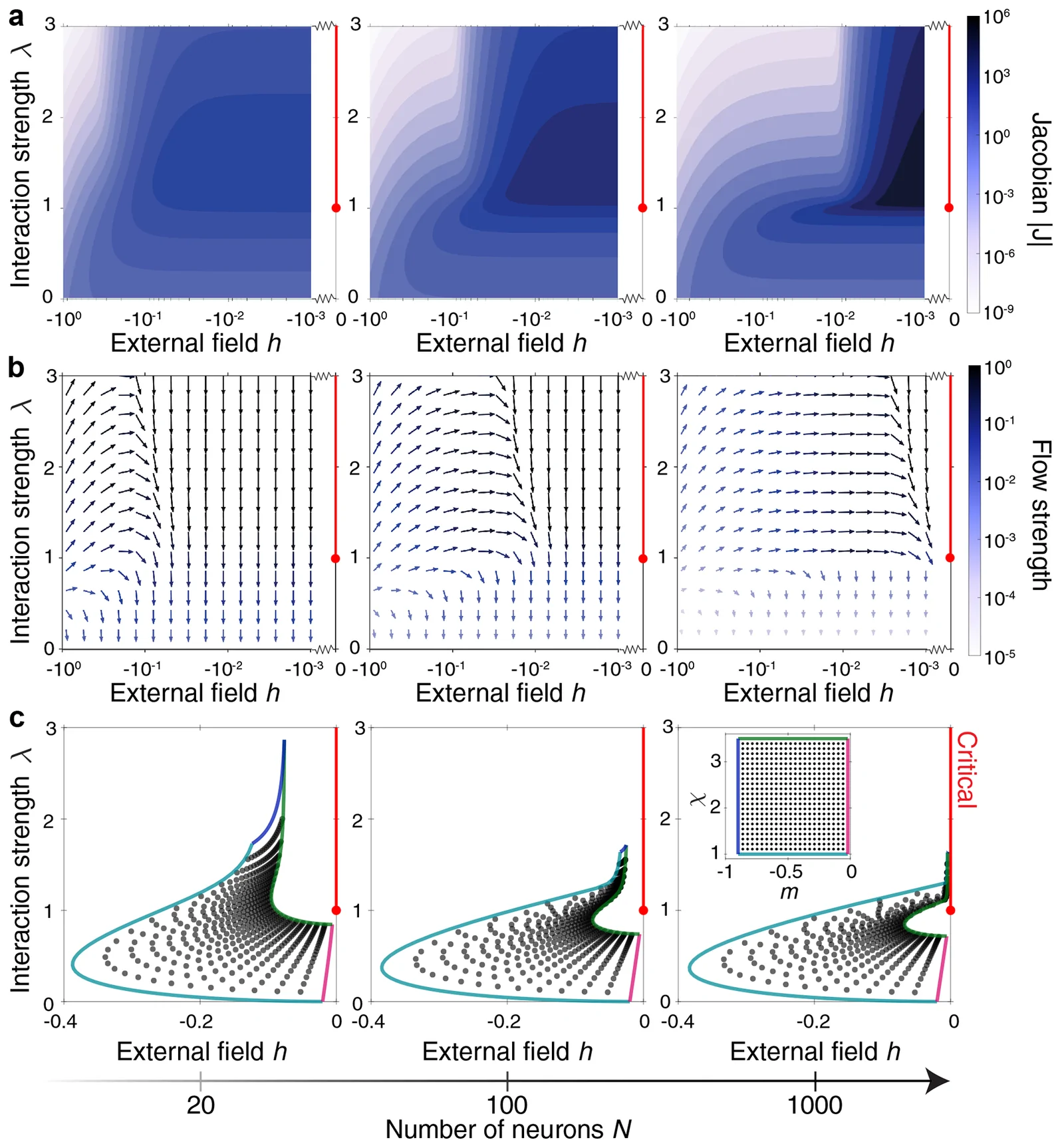
Large populations contract to critical models. **a**, Jacobian |*J*| as a function of the external field *h* and interaction strength λ. As the number of neurons increases, the Jacobian concentrates near the critical line. **b**, Flow in parameters due to a fractional increase in system size $\frac{d(h,\lambda )}{dlogN}$ with statistics (*m*, *χ*) held fixed (Methods). Flow magnitude is indicated by vector color and length. As *N* increases, many parameters flow toward the critical line. **c**, Inferred parameters corresponding to a fixed grid of statistics (*inset*). For larger populations, many statistics contract to critical models. Columns indicate populations of increasing size *N* = 20, 100, and 1000.

**Fig. 4 |:**
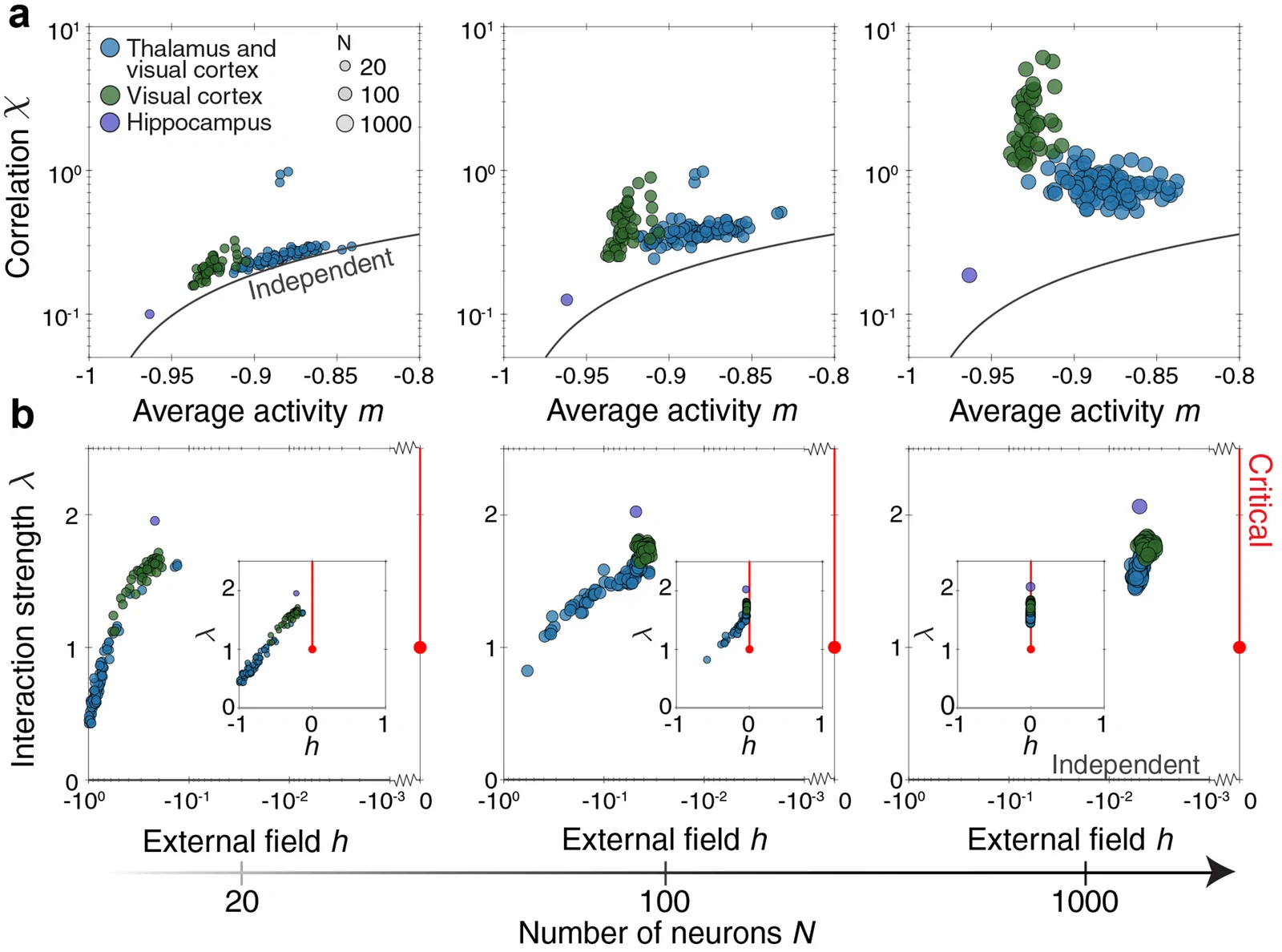
Models of neural activity flow toward criticality. **a**, Average activity *m* and correlation strength *χ* for subpopulations of increasing size *N* sampled from 146 recordings of neural activity from the thalamus and visual cortex (blue),^40, 41^ visual cortex (green),^42^ and hippocampus (purple).^43, 44^ Marker size indicates the number of neurons *N* in each subpopulation, and gray lines represent statistics that map to independent models. **b**, Inferred Ising parameters *h* and λ for each of the subpopulations in **a**. Red line defines the set of critical models in the thermodynamic limit *N* → ∞, and gray line represents independent models (λ = 0). Columns indicate populations of increasing size *N* = 20, 100, and 1000.

**Fig. 5 |:**
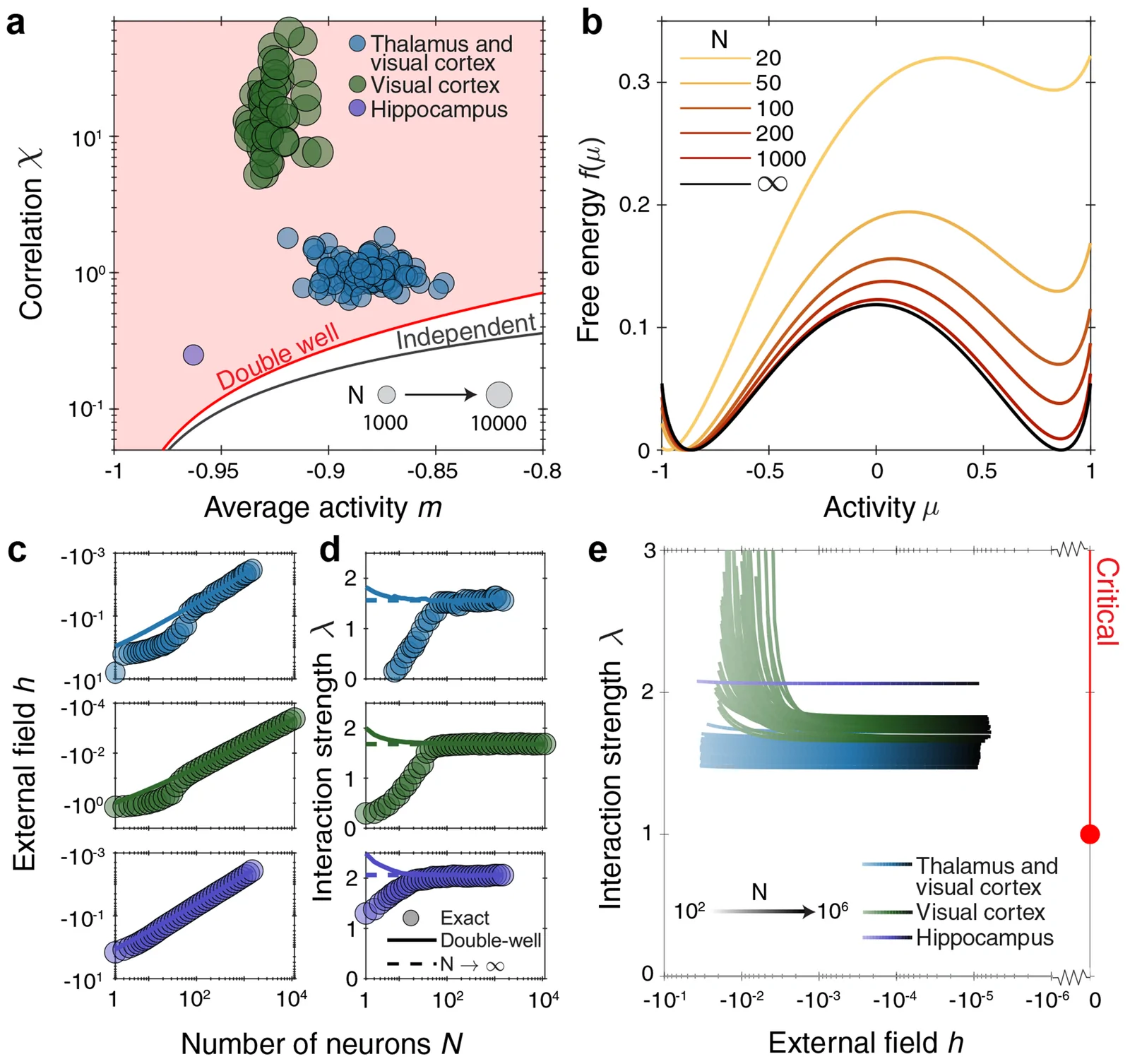
Collapse to criticality in the thermodynamic limit. **a**, Average activity *m* and correlation strength *χ* for the same recordings as Fig. 1. Red line defines the maximum correlations allowed under mean-field theory (Methods). Gray line defines statistics that map to independent models. Marker sizes indicate the number of neurons *N* in each population. **b**, Free energy *f*(*μ*) as a function of the population activity for subpopulations of increasing size sampled from the hippocampal recording.^43, 44^ See Methods for free energy expression. **c-d**, External field (**c**) and interaction strength (**d**) inferred for subpopulations of increasing size sampled from the thalamus and visual cortex (*top*),^40, 41^ visual cortex (*middle*),^42^ and hippocampus (*bottom*).^43, 44^ Markers represent exact parameters, solid lines represent the double-well approximation in Eq. (3), and dashed lines represent the thermodynamic limit of the double-well approximation. **e**, Double-well predictions for the external field and interaction strength [Eq. (3)] corresponding to the statistics in **a** with increasing population size *N*. As *N* → ∞, all recordings approach the critical line.

## Data Availability

The data analyzed in this paper are openly available at https://github.com/dpcarcamo/Emergence-of-Criticality.

## Code Availability

The code used to perform the analyses in this paper is openly available at https://github.com/dpcarcamo/Emergence-of-Criticality.
