# Supplementary material for: Emergence of criticality in models of real neurons
Source: ArXiv. 2026 Sep 8:arXiv:2609.09438v1. Preprint. [Version 1] (PMC13622868)
Supplement: Supplementary file 1 [file NIHPP2609.09438v1-supplement-1.pdf]

Supplementary Information

Emergence of criticality in models of real neurons

Contents

1 Introduction **2**

2 Bounds on statistics for finite system size **2**

3 Trajectories of inferred parameters with increasing system size **3**

4 Thermodynamic signatures of the inferred models **3**

5 Independent data **7**

References **9**

1 Introduction

In this Supplementary Information, we provide additional analyses that support and extend the results presented in the main text. In Sec. 2, we derive the finite-size bounds on the model statistics and identify the region of statistics space that can be realized by the Curie–Weiss model. In Sec. 3, we examine how inferred model parameters evolve with increasing system size and provide additional examples of the resulting trajectories through parameter space. In Sec. 4, we characterize the thermodynamic signatures of the inferred models using several complementary perturbations and measures of criticality. Finally, in Sec. 5, we study how the model behaves under independent data.

2 Bounds on statistics for finite system size

In the main text, we infer model parameters from points in statistics space. Although the parameters h and λ are unbounded, a finite system cannot realize arbitrary values of the mean activity m and correlation χ. For binary variables $x_i \in \{-1, 1\}$, the instantaneous population activity

$$\mu(\boldsymbol{x}) = \frac{1}{N} \sum_{i=1}^{N} x_i \tag{S1}$$

satisfies $-1 \leq \mu \leq 1$, and the mean activity is $m = \langle \mu \rangle$. Since

$$\chi = N \left(\langle \mu^2 \rangle - m^2 \right), \tag{S2}$$

the correlation is nonnegative. For a fixed mean m, the variance is maximized by a distribution supported at the endpoints $\mu = \pm 1$, with probabilities

$$P(\mu = \pm 1) = \frac{1 \pm m}{2}. \tag{S3}$$

Because $\langle \mu^2 \rangle = 1$ for this distribution, the maximum correlation is

$$\chi_{\max} = N \left(\langle \mu^2 \rangle - m^2 \right) \tag{S4}$$

$$= N \left(1 - m^2 \right). \tag{S5}$$

Thus, the finite-size statistics satisfy

$$-1 \leq m \leq 1, \qquad 0 \leq \chi \leq N \left(1 - m^2 \right). \tag{S6}$$

All points used in the fixed-statistics analyses were chosen to satisfy Eq. (S6) for every system size considered.

3 Trajectories of inferred parameters with increasing system size

The main text defines a flow field that describes how the inferred parameters (h, λ) change with system size while the statistics (m, χ) are held fixed. Here we integrate that flow to show complete parameter trajectories. Starting from an initial condition (m_0, χ_0, N_0), we increase N and follow the corresponding inferred parameters.

The trajectories reveal two regimes in statistics space (Fig. S1). Initial conditions in the red region, where the mean-field approximation discussed in the main text breaks down (Fig.5a), produce trajectories that approach the critical line $h = 0$, $\lambda > 1$. Outside this region, the trajectories approach distinct parameter values consistent with mean-field theory. These results complement the local flow field in Fig.3b by showing the integrated paths of inferred models as N increases.

4 Thermodynamic signatures of the inferred models

For each neural dataset and population size, we inferred the Curie–Weiss parameters (h, λ) from the measured mean activity m and correlation χ. Because these parameters define an exact finite-size model, we can perturb the inferred model and study the response of the statistics (Fig. S2).

We first introduce a fictitious temperature T by rescaling both inferred parameters,

$$h(T) = \frac{h}{T}, \qquad \lambda(T) = \frac{\lambda}{T}. \tag{S7}$$

The original inferred model is recovered at $T = 1$. A peak in the specific heat $C(T)/N$, where $C(T)$ is the heat capacity, is a signature that an inferred maximum-entropy model is poised near a second-order phase transition.[1] In our models, the maximum of $C(T)/N$ occurs at $T > 1$ (Fig. S2a). This reflects the fact that the inferred models lie near a first-order, rather than second-order, transition.

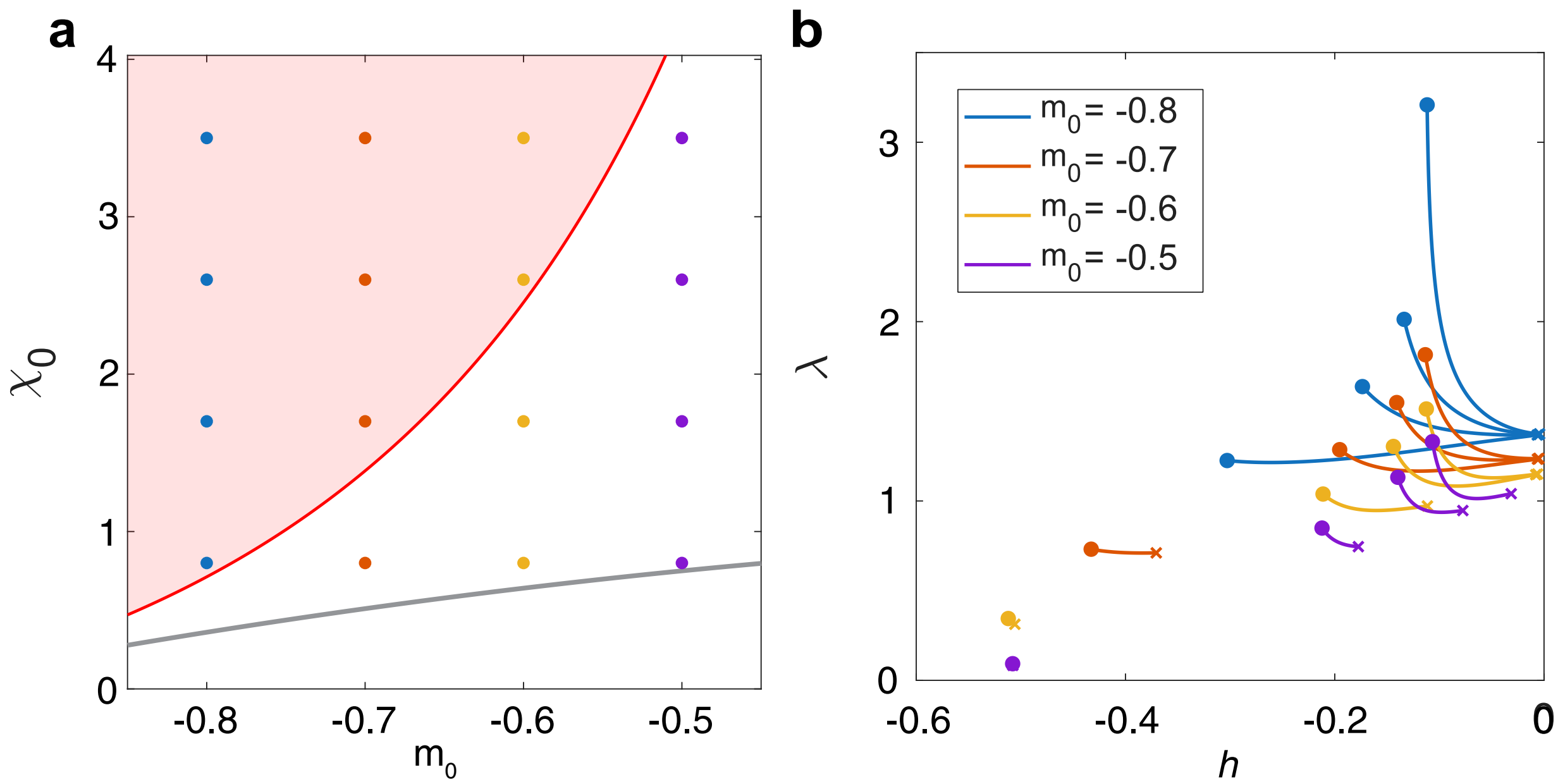

Fig. S1 | **Trajectories of inferred parameters as system size increases at fixed statistics. a**, Initial conditions in statistics space. Color indicates the mean activity m_0. The red region marks statistics for which the mean-field approximation breaks down, as shown in Fig.5a. **b**, Parameter trajectories obtained by integrating the flow from the initial conditions in **a**, beginning at $N_0 = 10$. Initial conditions in the red region generate trajectories that approach the critical line, whereas points outside this region approach distinct parameter values consistent with the mean-field theory.

We next perturb the interaction strength according to

$$\lambda(\alpha) = \alpha\lambda, \tag{S8}$$

with $\alpha = 1$ corresponding to the inferred model, and choose $h(\alpha)$ so that the mean activity m remains fixed. This perturbation removes the change in mean activity that accompanies the fictitious temperature. Along this fixed-mean path, the specific heat is maximized at $\alpha = 1$ (Fig. S2b).

The same distinction between the two different parameter sweeps appears in the Jacobian $J = \partial(m, \chi)/\partial(h, \lambda)$. Along the fictitious-temperature path, $|J(T)|$ is maximized at $T > 1$ (Fig. S2c). By contrast, along the fixed-mean path, $|J(\alpha)|$ peaks at $\alpha = 1$ (Fig. S2d). This result directly supports the geometric interpretation developed in the main text: when pertur-

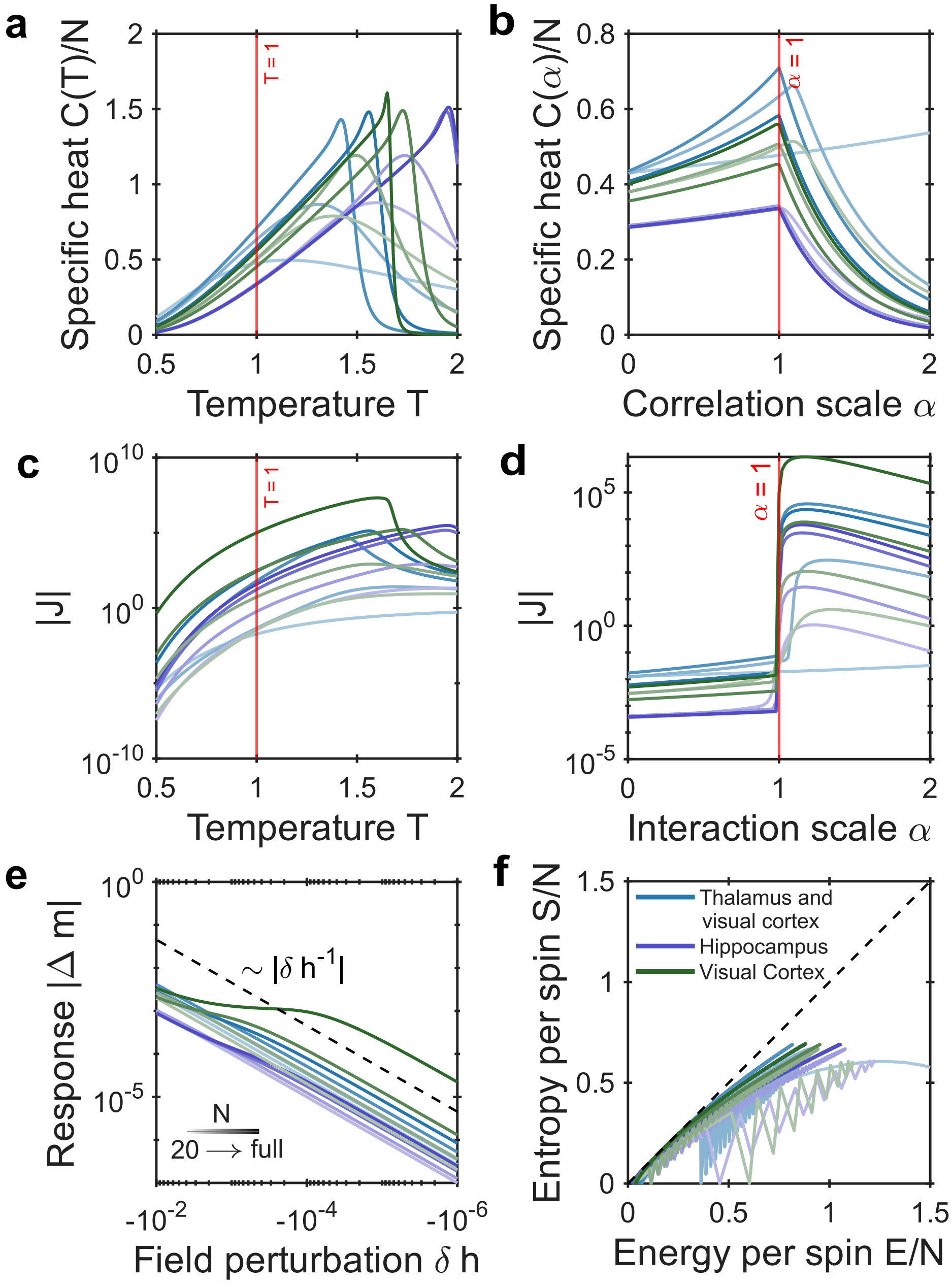

Fig. S2 | **Thermodynamic signatures of the inferred Curie–Weiss models.** The three populations studied in the main text are plotted in blue, green, and purple for the thalamus and visual cortex, visual cortex, and hippocampus data, respectively. The population sizes are $N = 20$, 100, 1000, and the full population. **a**, Specific heat, $C(T)/N$, along the fictitious-temperature path $(h/T, \lambda/T)$. The peak lies near, but not exactly at, the inferred model $T = 1$. **b**, Specific heat along the fixed-mean path, with $\lambda(\alpha) = \alpha\lambda$ and $h(\alpha)$ chosen to preserve m. Along this path, the inferred model at $\alpha = 1$ lies at the point of maximal sensitivity. **c**, Magnitude of the Jacobian determinant, $|J(T)|$, along the fictitious-temperature path. The inferred model lies close to, but slightly displaced from, the maximum.

d, Magnitude of the Jacobian determinant along the fixed-mean path, which is maximized at $\alpha = 1$. **e**, Change in mean activity, Δm, produced by perturbing the inferred external field h while holding λ fixed. Solid curves show the response of the models, and the dotted curve shows the predicted scaling of the response as a function of the external field from the double-well approximation. **f**, Entropy per neuron versus energy per neuron for the inferred models. The approach to a unit-slope relation indicates that the increasing number of higher-energy states compensates for their decreasing probability. In **a–d**, red vertical lines mark the unperturbed model, corresponding to $T = 1$ or $\alpha = 1$.

bations are constrained to preserve the measured mean activity, the inferred model lies at a point where small parameter changes produce especially large changes in the remaining model statistics.

Fig. S2e tests the response of the inferred models to perturbations of the external field. For each inferred model, we vary h around its inferred value while holding λ fixed and calculate the resulting change in mean activity, Δm. The solid curves show the responses calculated from models with different population sizes. The dotted line shows the predicted scaling from the double-well approximation derived in the main text. Their agreement shows that the strong response of the mean activity to small field perturbations is captured by the double-well approximation to the free energy near the critical line.

Finally, we compare entropy and energy across the inferred models (Fig. S2f). In an equilibrium representation, the number of states at energy E grows as $\exp[S(E)]$, while their Boltzmann weight at $T = 1$ decreases as $\exp(-E)$. The competition between these terms selects a narrow range of typical energies. If instead $S(E)$ approaches a line with unit slope, the growth in the number of states compensates for the decrease in their probability over a broad energy range. This entropy–energy balance was identified as a signature of criticality in neural maximum-entropy models.[1] The inferred Curie–Weiss models approach this relation as population size increases, providing an additional indication that they lie close to a critical thermodynamic structure. Together, Fig. S2a-f show that the response of the system depends on the direction in which the inferred model is perturbed, consistent with the proximity to a

first-order phase transion observed in the main text.

5 Independent data

To determine how correlations in the neural activity affect the inferred models, we shuffle the time series of each neuron independently. This procedure preserves the mean activity of each neuron while removing correlations between neurons in expectation. We then calculate m and χ from the shuffled time series and infer the corresponding Curie-Weiss parameters h and λ in the same way as for the original data. Shuffling moves the measured statistics decreases the total correlation χ and correspondingly moves the inferred models toward $\lambda = 0$ (Fig. S3).

Since the shuffled time series have finite length, however, the measured pairwise correlations do not vanish exactly. These finite-sampling fluctuations can cause some shuffled datasets to lie above the independent Curie–Weiss curve, $\chi = 1 - m^2$. To remove this finite-sampling effect, we therefore considered a second control in which we assumed exact independence and calculated χ directly from the measured mean activity of each neuron.

For independent neurons, all off-diagonal covariances vanish, giving

$$\chi = \frac{1}{N} \sum_i \left(\langle x_i^2 \rangle - \langle x_i \rangle^2 \right) = 1 - \frac{1}{N} \sum_i \langle x_i \rangle^2, \tag{S9}$$

where we have used $x_i^2 = 1$. Defining the average squared deviation of the individual neuron means from the population mean as

$$\Delta_x^2 = \frac{1}{N} \sum_i \left(\langle x_i \rangle - m \right)^2, \tag{S10}$$

and using $m = N^{-1} \sum_i \langle x_i \rangle$, we have

$$\frac{1}{N} \sum_i \langle x_i \rangle^2 = m^2 + \Delta_x^2. \tag{S11}$$

The correlation of an independent population is therefore

$$\chi = 1 - m^2 - \Delta_x^2. \tag{S12}$$

Since $\Delta_x^2 \geq 0$, an independent population with heterogeneous mean activities $\langle x_i \rangle$ must satisfy

$$\chi \leq 1 - m^2, \tag{S13}$$

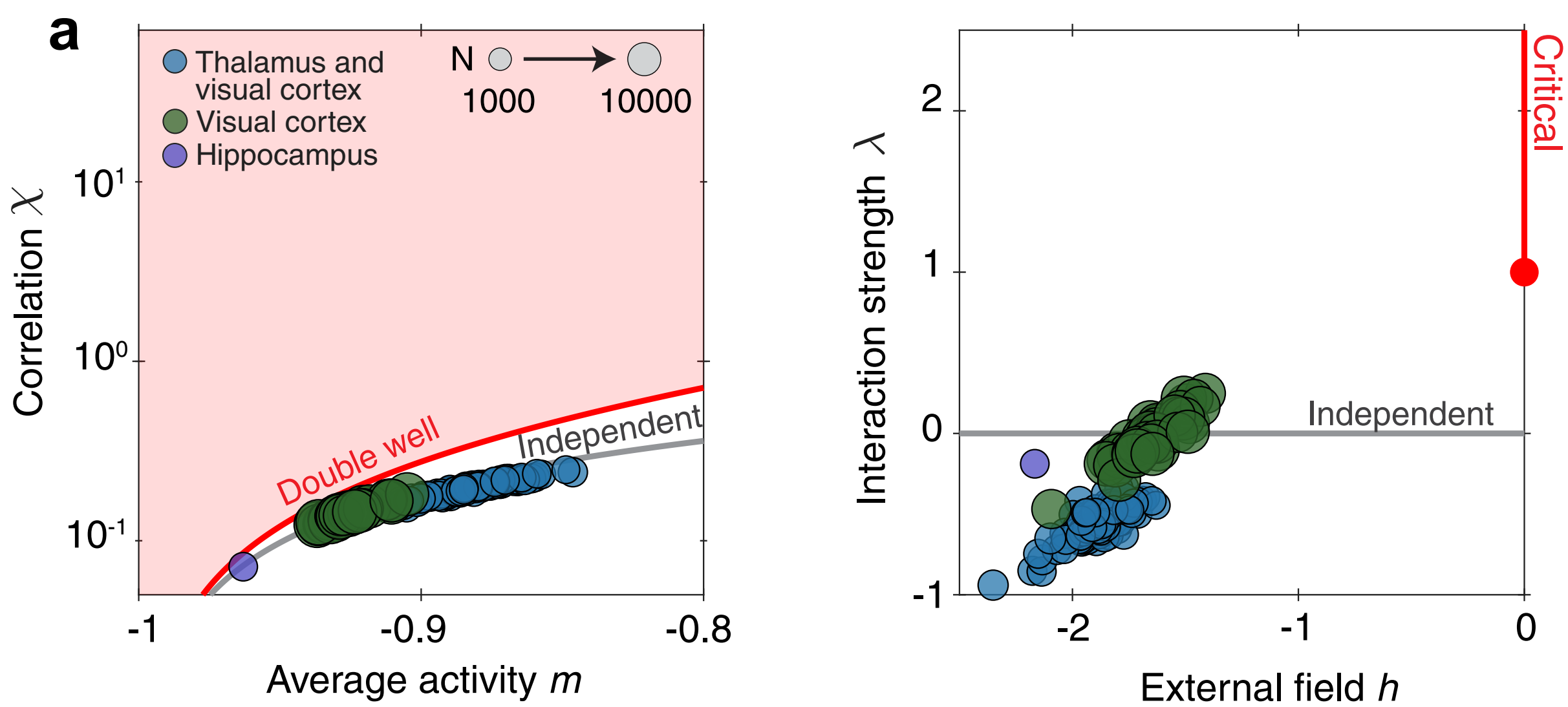

Fig. S3 | **Shuffled data.** The time series of each neuron was independently shuffled, preserving the mean activity of each neuron while removing correlations between neurons in expectation. Left, the measured m and χ for the shuffled data. The gray line shows the statistics of the independent Curie–Weiss model ($\lambda = 0$), $\chi = 1 - m^2$. Shuffling moves the measured statistics toward the region expected for independent activity. Because the shuffled time series have finite length, residual correlations due to finite sampling can place some points above the independent curve. Right, the corresponding Curie–Weiss parameters h and λ inferred from the shuffled statistics. Shuffling moves the inferred models toward the non-interacting model, $\lambda = 0$.

with equality only when all neurons have the same mean activity. When exact independence is imposed, all of the datasets therefore lie at or below the independent Curie-Weiss curve (Fig. S4). When inferring Curie-Weiss models, this yields non-positive interactions $\lambda \leq 0$.

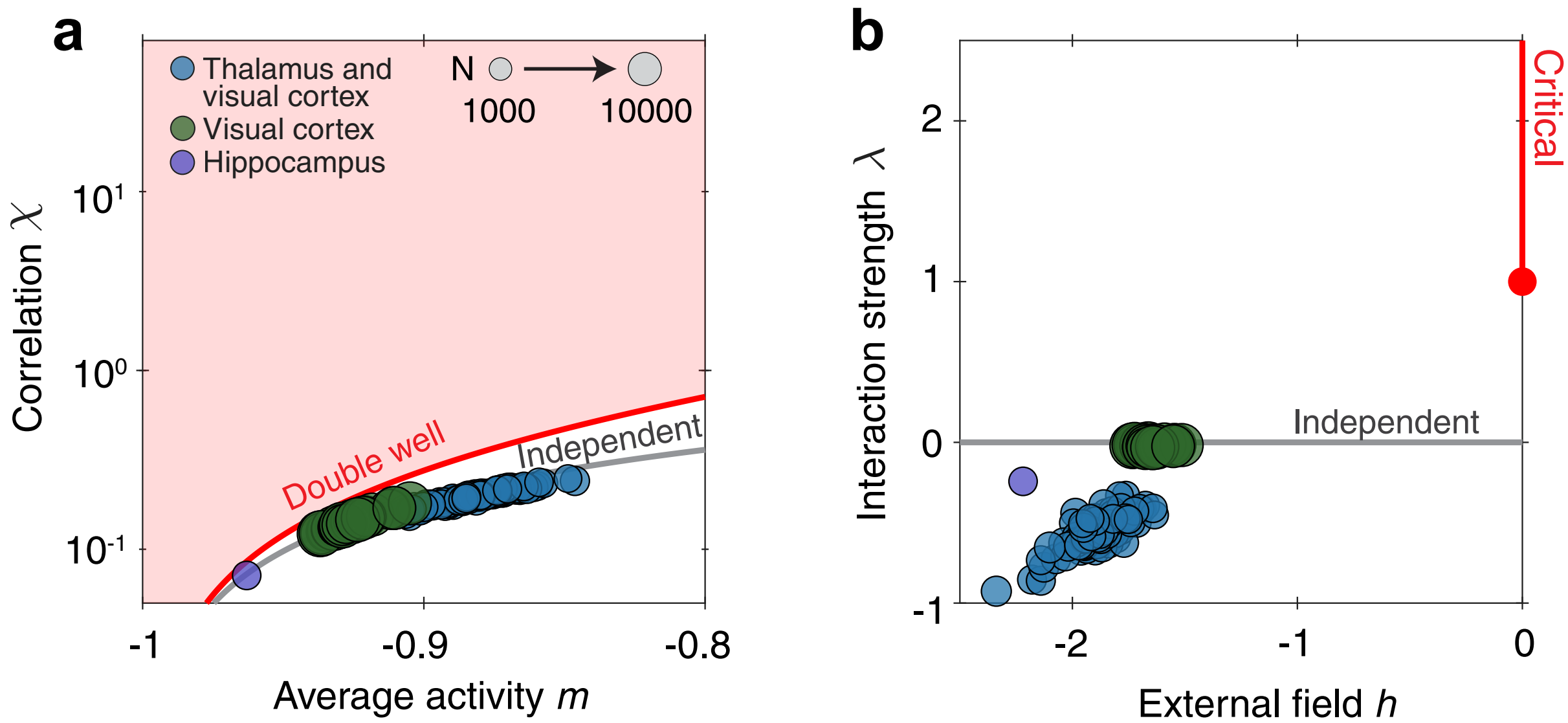

Fig. S4 | **Independent data.** The statistics expected under exact independence were calculated directly from the measured mean activity of each neuron, with all off-diagonal covariances set to zero. Left, the resulting m and χ. The gray line shows the independent Curie–Weiss model ($\lambda = 0$), $\chi = 1 - m^2$. Heterogeneity in the mean activity of individual neurons reduces the correlation to $\chi = 1 - m^2 - \Delta_x^2$, where $\Delta_x^2 = N^{-1} \sum_i (\langle x_i \rangle - m)^2$. The statistics therefore lie at or below the independent Curie–Weiss curve when finite-sampling correlations are removed. Right, the corresponding h and λ obtained by mapping these statistics onto the Curie–Weiss model.

References

1. Tkačik, G. *et al.* Thermodynamics and signatures of criticality in a network of neurons. *Proc. Natl Acad. Sci. USA* **112**, 11508–11513 (2015).
